# Pathological shifts in tryptophan metabolism in human term placenta exposed to LPS or poly I:C^†^

**DOI:** 10.1093/biolre/ioad181

**Published:** 2023-12-25

**Authors:** Cilia Abad, Rona Karahoda, Anna Orbisova, Petr Kastner, Daniel Heblik, Radim Kucera, Ramon Portillo, Frantisek Staud

**Affiliations:** Department of Pharmacology and Toxicology, Faculty of Pharmacy in Hradec Kralove, Charles University, Hradec Kralove, Czech Republic; Department of Pharmacology and Toxicology, Faculty of Pharmacy in Hradec Kralove, Charles University, Hradec Kralove, Czech Republic; Department of Pharmacology and Toxicology, Faculty of Pharmacy in Hradec Kralove, Charles University, Hradec Kralove, Czech Republic; Department of Pharmaceutical Chemistry and Pharmaceutical Analysis, Faculty of Pharmacy in Hradec Kralove, Charles University, Hradec Kralove, Czech Republic; Department of Pharmaceutical Chemistry and Pharmaceutical Analysis, Faculty of Pharmacy in Hradec Kralove, Charles University, Hradec Kralove, Czech Republic; Department of Pharmaceutical Chemistry and Pharmaceutical Analysis, Faculty of Pharmacy in Hradec Kralove, Charles University, Hradec Kralove, Czech Republic; Department of Pharmacology and Toxicology, Faculty of Pharmacy in Hradec Kralove, Charles University, Hradec Kralove, Czech Republic; Department of Pharmacology and Toxicology, Faculty of Pharmacy in Hradec Kralove, Charles University, Hradec Kralove, Czech Republic

**Keywords:** intrauterine infections, placenta, tryptophan metabolism, fetal brain development, programming

## Abstract

Maternal immune activation during pregnancy is a risk factor for offspring neuropsychiatric disorders. Among the mechanistic pathways by which maternal inflammation can affect fetal brain development and programming, those involving tryptophan (TRP) metabolism have drawn attention because various TRP metabolites have neuroactive properties. This study evaluates the effect of bacterial (lipopolysaccharides/LPS) and viral (polyinosinic:polycytidylic acid/poly I:C) placental infection on TRP metabolism using an ex vivo model. Human placenta explants were exposed to LPS or poly I:C, and the release of TRP metabolites was analyzed together with the expression of related genes and proteins and the functional activity of key enzymes in TRP metabolism. The rate-limiting enzyme in the serotonin pathway, tryptophan hydroxylase, showed reduced expression and functional activity in explants exposed to LPS or poly I:C. Conversely, the rate-limiting enzyme in the kynurenine pathway, indoleamine dioxygenase, exhibited increased activity, gene, and protein expression, suggesting that placental infection mainly promotes TRP metabolism via the kynurenine (KYN) pathway. Furthermore, we observed that treatment with LPS or poly I:C increased activity in the kynurenine monooxygenase branch of the KYN pathway. We conclude that placental infection impairs TRP homeostasis, resulting in decreased production of serotonin and an imbalance in the ratio between quinolinic acid and kynurenic acid. This disrupted homeostasis may eventually expose the fetus to suboptimal/toxic levels of neuroactive molecules and impair fetal brain development.

## Teaser

LPS and poly I:C impair tryptophan metabolism in the human placenta explants.

## Graphical Abstract

**Graphical Abstract:**
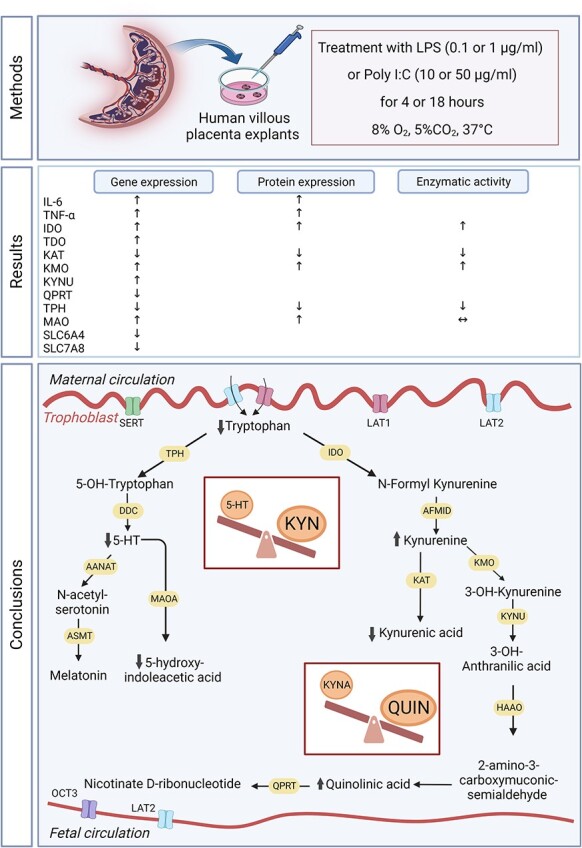

## Introduction

Intrauterine inflammation occurs in approximately 20% of all pregnancies and an alarming 85% of pregnancies leading to very premature births [1, 2]. Several lines of evidence including epidemiological data and results from animal studies clearly indicate that maternal inflammation during pregnancy is linked to increased risks of neurodevelopmental and psychiatric disorders in offspring [3]. Specifically, reports have shown an increased risk of motor-sensory deficits, impaired working memory abilities, delayed learning, and neurological diseases including schizophrenia, autism spectrum disorder, and epilepsy [1, 4].

The fact that several different infections in pregnant women are associated with identical risks of neurodevelopmental disorders in offspring indicates that it is the maternal immune response or maternal inflammation rather than the pathogen itself that is responsible for the link between prenatal infections and neurodevelopmental disorders [5]. Intrauterine infection is typically detected by the maternal innate immune system [6], which induces downstream synthesis and enhanced maternal circulation of pro-inflammatory cytokines such as IL-6, IL-8, IL-1β, TNF, and macrophage inflammatory proteins [7]. Furthermore, elevated levels of cytokines IL-6 and IL-1β in amniotic fluid and placental inflammation predict brain injury in premature infants [1, 8].

Although epidemiological data strongly support an association between maternal inflammation and fetal neurodevelopment disorders [9], the mechanistic links between these processes remain unclear. Among the mechanistic pathways by which maternal inflammation during pregnancy can affect fetal brain development and programming, the role of tryptophan (TRP) metabolism has received considerable attention because various TRP metabolites have neuroactive properties [10, 11]. TRP is an essential amino acid in many physiological processes including protein synthesis and for healthy placental and fetal development [12]. Two main TRP metabolic pathways known as the kynurenine (KYN) and serotonin (5-HT) pathways have been identified in several organs, including the placenta. The relative flux through these pathways depends on the physiological stage of pregnancy [10].

TRP catabolism through the KYN pathway generates several metabolites with neuroactive, antioxidant, and immunoregulatory functions [13]. The first and rate-limiting enzyme in this pathway, indoleamine 2,3-dioxygenase (IDO), exhibits increased gene- and protein-level expression along with increased functional activity as the placenta develops [14, 15] and is strongly induced by several pro-inflammatory cytokines including interferon-γ (IFN-γ) [16]. The downstream catabolism of TRP after conversion into KYN diverges into two main branches: the aminotransferase (KAT) enzyme branch, which leads to the formation of the neuroprotective agent kynurenic acid (KYNA), and the KYN monooxygenase (KMO) enzyme branch, leading to three hydroxy-kynurenine (3-OH-KYN) and the neurotoxic quinolinic acid (QUIN). In addition to being formed via different branches of the KYN pathway, KYNA and QUIN have opposing effects on NMDA receptors: KYNA is an antagonist, while QUIN is an agonist. Maintaining the balance between these two metabolites is crucial for proper glutamatergic neurotransmission [17]. Any shift in the ratio of QUIN to KYNA can have severe consequences, potentially causing neurodevelopmental disorders in the offspring. Furthermore, these placenta-derived KYN metabolites have been implicated in the etiology of perinatal brain damage [18].

TRP metabolism via the 5-HT pathway produces active metabolites such as 5-HT and melatonin [19]. The key enzymes of this pathway are tryptophan hydroxylase (TPH), which is the pathway’s first and rate-limiting enzyme, and monoamine oxidase (MAO), which degrades 5-HT [20, 21]. Placental synthesis of 5-HT is particularly important for successful blastocyst implantation, placentation, and decidualization in the early stages of pregnancy [22, 23]. Moreover, placenta-derived 5-HT is crucial for fetal brain development [24]. Within the placenta, some 5-HT may be transformed into melatonin [25], which is involved in fetal growth and regulating placental function [26, 27]. Since the placenta is an essential source of 5-HT for the fetal brain, perturbations in placental TRP metabolism may disrupt 5-HT signaling in specific regions of the developing fetal brain, leading to abnormal programming of major axonal pathways [21].

Inflammatory insults modulate both the KYN and the 5-HT metabolic pathways; experimental studies have shown that pro-inflammatory cytokines induce the expression of several enzymes involved in tryptophan catabolism [28]. Furthermore, animal models of systemic inflammation or intrauterine infection have demonstrated alterations in the gene expression of key enzymes within the 5-HT pathway [29, 30]. In women, intrauterine infections are associated with upregulated expression of genes encoding KYN pathway enzymes in the placenta [31]. We recently demonstrated that preterm birth is associated with significant changes in placental TRP metabolism gene expression and that this effect correlates positively with levels of intraamniotic and maternal inflammatory markers [32]. In this work, we hypothesize that bacterial (lipopolysaccharides/LPS) or viral (polyinosinic:polycytidylic acid/poly I:C) infection may impair TRP metabolism in the placenta, triggering a shift in tryptophan metabolism toward the KYN pathway that alters the QUIN/KYNA ratio and thereby creates a neurotoxic environment in the fetoplacental unit.

Using an ex vivo model of human placenta explants, we aimed to examine the impact of bacterial (LPS) and viral (poly I:C) infections on TRP metabolism in the human placenta. To this end, we comprehensively analyzed the expression of genes encoding key enzymes in TRP metabolism via the KYN and 5-HT pathways as well as the expression and functional activity of the corresponding proteins. We also quantified TRP and its metabolites in the supernatant media from inflamed placenta samples.

## Methods

### Chemicals and reagents

Lipopolysaccharides from *Escherichia coli* O111:B4 (LPS), polyinosinic–polycytidylic acid sodium salt (poly I:C), and thiazolyl blue tetrazolium bromide (MTT) were purchased from Sigma-Aldrich (St. Louis, MO, USA). Bicinchoninic acid assay (BCA assay) reagents were purchased from Thermo Scientific (Rockford, IL, USA). Tri Reagent solution was obtained from the Molecular Research Centre (Cincinnati, OH, USA). All other chemicals were of analytical grade.

### Human placenta sample collection

Human term placentas were collected from pregnant women at 38–40 weeks of gestation undergoing elective caesarean section delivery with no pregnancy complications. Placentas were obtained immediately after delivery at the University Hospital in Hradec Kralove, Czech Republic. All experiments were performed in accordance with the Declaration of Helsinki, and human placenta samples were obtained with the women’s written informed consent and the approval of the University Hospital Research Ethics Committee (201006 S15P).

### Explant culture

**Figure 1:**
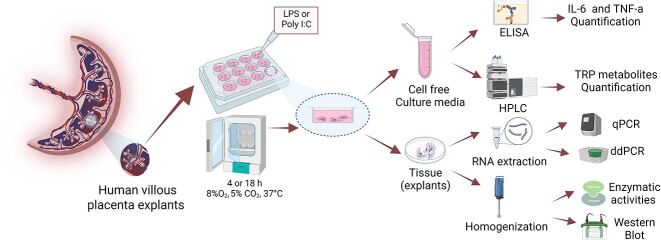
Experimental design used in this study. Healthy term placenta explants were prepared and cultured in 12-well plates for a stabilization period of 24–36 h. The explants were then incubated with either LPS (0.1 or 1 μg/ml) or poly I:C (10 or 50 μg/ml) for 4 or 18 h. Cell-free culture media (supernatant) were collected to quantify pro-inflammatory cytokines using ELISA and TRP and its metabolites using HPLC. Explant tissue was also used for qPCR, ddPCR, and homogenates for western blot and functional analysis. Created in BioRender.com.

Cotyledon fragments were gently separated by dissection from different areas of each placenta and the chorionic plate and decidua were removed. Villous tissue was further dissected into explants of approximately 30 mg. Randomly sampled villous tissue was cleaned of large vessels and blood clots, rinsed with cold sterile saline, and placed in 12-well plates containing a culture medium consisting of 2 ml of DMEM-F12 medium and 10% fetal calf serum enriched with 100 U/ml penicillin, 0.1 mg/ml streptomycin, and 2.5 𝜇g/ml amphotericin B [33]. Three explants were placed in each well, giving roughly 100 mg of explanted tissue per well. The villous explants were incubated under an atmosphere of 8% O_2_, 5% CO_2_, and 87% N_2_ at 37°C in a sterile incubator for 4 h to equilibrate the cultures and allow recovery from the isolation procedure. The explants were kept in these culture conditions for 24–36 h before experimentation. They were then incubated with LPS (0.1 or 1 μg/ml) or poly I:C (10 or 50 μg/ml) for 4 or 18 h. After incubation, cell-free supernatant was collected for analysis. Snap-frozen tissue samples and homogenates were prepared from the tissue. The research design and experimental approaches employed in this study are shown in Figure 1.

### Analysis of placental explants’ viability and integrity

Explant viability was determined after 4 and 18 h treatments using the MTT (thiazolyl blue tetrazolium bromide) reduction assay [34]. To determine the incorporation of MTT into the tissue, explants were washed with Opti-MEM^TH^ and incubated with 0.5 mg/ml MTT solution at 37°C for 1 h before being transferred to a new well containing 1 ml DMSO and incubated for 5 min with shaking at room temperature. The quantity of formazan in the well was then determined by monitoring the supernatant’s relative absorbance (Abs) at wavelengths of 570 and 690 nm. As positive controls, explants cultured for 18 h with 40% DMSO were analyzed in the same way. Results are expressed as the difference between Abs 570 and Abs 690 per gram of tissue. Explant integrity was verified by measuring the release of the intracellular enzyme lactate dehydrogenase (LDH) into the incubation medium using a colorimetric LDH activity assay kit from Sigma-Aldrich (St. Louis, MO, USA) following the manufacturer’s instructions [35]. LDH activity in the culture media was normalized against the explant mass (milligrams), and the results obtained were expressed as nanomoles of NADH/ml*min/mg tissue. As positive controls, additional experiments were performed in which the same assay was performed using samples for which LDH release had been maximized by culturing explants in lysis buffer (20 mM Tris-HCl, 150 mM NaCl, 12.7 mM EDTA, 1 mM EGTA, 4 mM Na_4_P_2_O_7_, 1 mM Na_3_VO_4_, 1% Triton X-100, and protease inhibitors cocktail, pH 6.8) for 15 min at 37°C.

### Cytokine analysis

Protein levels of the pro-inflammatory cytokines IL-6 and TNF-α were measured using a highly sensitive enzyme-linked immunosorbent assay (ELISA) from Thermo Scientific (Rockford, IL, USA) according to the manufacturer’s protocols.

### RNA isolation and reverse transcription

Total RNA was isolated from 100 mg of placental explant tissue using Tri Reagent solution, according to the manufacturer’s instructions. The purity of the isolated RNA was verified by measuring the A260/A280 ratio and contamination by organic solvents was evaluated using the A260/230 ratio. Absorbance ratios were measured using a NanoDrop 1000 Spectrophotometer (Thermo Fisher Scientific, Waltham, MA, USA). The absorbance at 260 nm was used to calculate the total RNA concentration. Reverse transcription (RT) was performed using the iScript Advanced cDNA Synthesis Kit and T100 Thermal Cycler (Bio-Rad, Hercules, CA, USA).

### Quantitative PCR analysis

Quantitative PCR (qPCR) analysis of gene expression was performed using QuantStudio 6 (Thermo Fisher Scientific, Waltham, MA, USA). cDNA (12.5 ng/μl) was amplified using the TaqMan Universal Master Mix II without UNG (Thermo Fisher Scientific, Waltham, MA, USA) in a total reaction volume of 5 μl/well with predesigned TaqMan Real Time Expression PCR assays, following the thermal program specified in the manufacturer’s instructions. The relative gene expression was normalized against the geometric mean expression of the reference genes Ubiquitin *(UBC)*, DNA Topoisomerase I (*TOP1*), Eukaryotic Translation Initiation Factor 4A2 *(EIF4A2)*, tyrosine 3-monooxygenase/tryptophan 5-monooxygenase activation protein zeta (*YWHAZ*), and β2 microglobulin (*B2M*). The normalized expression values were then used to generate a gene expression heat map with the freely available Heatmapper web server (http://www.heatmapper.ca/).

### Droplet digital PCR

Duplex droplet digital PCR (ddPCR) analysis of *IDO, KAT1, KMO, TPH,* and *MAO* in placental explants was performed as described previously [15]. The duplex feature allowed us to absolutely quantify the expression of target and reference genes simultaneously. Briefly, each duplex reaction mixture consisted of 10 μl of ddPCR Supermix for Probes (Bio-Rad, Hercules, CA, USA), 1 μl of each of the predesigned probe assays (FAM and HEX), and 1 μl of cDNA (25 ng/μl) in a total volume of 20 μl. Droplets were generated using a QX200 Droplet Generator and amplified to end-point using a T100 Thermal Cycler using the thermal program specified in the manufacturer’s instructions. Droplet counting was performed with a QX200 Droplet Reader, and the target gene’s concentration was calculated using the QuantaSoft Software. For final data evaluation, only wells with droplet numbers above 13 000 were included. Results are reported as numbers of transcripts/ng of transcribed RNA. The QX200 Droplet Digital PCR System, T100 Thermal Cycler, and all consumables and reagents were obtained from BioRad (Hercules, CA, USA) unless otherwise stated.

### Placenta homogenates

Explant tissues were washed with 0.9% NaCl at 4°C. After weighing, explants were cut into small pieces and homogenized at 4°C in a buffer containing 50 mM Tris-Hepes (pH 7.2), 5 mM EGTA, 5 mM EDTA, 1 mM phenylmethylsulfonyl fluoride, and 250 mM sucrose. The homogenates were centrifuged at 10 000*g* for 15 min. The supernatant was collected and stored in the freezer at −80°C until use. Protein concentration was determined using a Pierce BCA protein assay kit according to the manufacturer’s instructions.

### Monoamine oxidase enzymatic activity

Monoamine oxidase (MAO) activity was determined by the method of Carrasco et al. [36]. Briefly, 180 μl placenta homogenate (1.5–2 mg/ml) was preincubated for 5 min at 37°C, with or without phenelzine (100 μM), and the reaction was initiated by incubation with 20 μl of 5-HT (0.5 mM) for 60 min. The reaction was then stopped by adding 40 μl of HClO_4_ (3.4 M) and placed on ice for 5 min. Samples were centrifuged at 5000*g* for 10 min, after which the supernatant was collected and used for 5-HT determination by high-performance liquid chromatography (HPLC). MAO activity is expressed as metabolized 5-HT (% of initially added 5-HT).

### Indoleamine 2,3-dioxygenase enzymatic activity

Indoleamine 2,3-dioxygenase (IDO) activity was determined as described by Takikawa et al. [37]. Samples were incubated for 5 min at 37°C in incubation media (400 μl) containing 50 mM potassium phosphate buffer pH 6.5, 20 mM ascorbate, 0.01 mM methylene blue, and 100 units/ml catalase, with or without 0.4 mM L-tryptophan. The reaction was initiated by adding the enzyme suspension (100 μl placenta homogenate) and terminated after 30 min by adding 100 μl of 30% trichloroacetic acid, after which the mixture was incubated at 50°C for 30 min to hydrolyze the N-formyl kynurenine produced by IDO into kynurenine. The reaction mixture was then centrifuged at 3000*g* for 20 min at 20°C, and 200 μl supernatant was collected for KYN determination by HPLC. IDO activity was calculated as the difference between the amount of KYN produced in media containing L-tryptophan and that produced in media without L-tryptophan. Indoleamine 2,3-dioxygenase activity is expressed as nanomoles of kynurenine per microgram of protein per minute.

### Tryptophan hydroxylase enzymatic activity

For determination of tryptophan hydroxylase (TPH) enzymatic activity, placenta homogenates were supplemented with 1 mM dithiothreitol (DTT) to reduce the protein and maximize its enzymatic activity [38]. TPH activity was determined by the method described by Goeden et al. [29]. The enzymatic reaction was carried out by incubating 100 μl of placental explant homogenate for 30 min at 37°C and pH 7.5 in 400 μl of incubation media containing (final concentrations) 50 mM Tris buffer, 1 mM EGTA, 100 units/ml catalase, 0.1 mM ammonium iron (II) sulfate, and 0.1 mM tetrahydrobiopterin (BH4, a cofactor required for TPH activity), with or without 0.25 mM L-tryptophan. Reactions were terminated by adding 100 μl of HClO_4_ with 100 μM EDTA, after which samples were incubated on ice for 15 min to ensure complete protein denaturation and then centrifuged at 21 000*g* for 15 min. Supernatants were collected for 5-OH-tryptophan determination by HPLC. TPH activity was calculated as the difference between the amounts of 5-OH-tryptophan liberated in tubes with and without L-tryptophan and is expressed as nanomoles of 5-OH-tryptophan per milligram of protein per minute.

### Kynurenine monooxygenase enzymatic activity

Kynurenine monooxygenase (KMO) in placental homogenates was determined by using HPLC to measure the conversion of KYN to 3-OH-KYN according to a slightly modified variant of the protocol described by Notarangelo et al. [39]. Briefly, 120 μl of incubation media containing 1 mM NADPH, 10 mM KCl, 1 mM EDTA, 100 mM Tris–HCl pH 8.1, and 0.5 mM ascorbic acid with or without 125 μM KYN were incubated with 80 μl of placental explant homogenate at 37°C for 60 min. The reaction was terminated by adding 50 μl of 6% HClO_4_ and incubating on ice for 5 min. Samples were centrifuged at 15 000*g* for 15 min, and 200 μl of supernatant was collected for 3-OH-KYN analysis by HPLC. KMO activity was then calculated as the difference between the amounts of 3-OH-KYN produced in tubes with and without KYN and is expressed as nanomoles of 3-OH-KYN per milligram of protein per hour.

### Kynurenine aminotransferase enzymatic activity

Placental kynurenine aminotransferase (KAT) activity was assayed using the protocol of Milart et al. [40] with modifications. Briefly, 80 μl of placental homogenate was incubated at 37°C for 60 min in 160 μl of incubation media containing 150 mM Tris-Acetate pH 9.8, 70 μM pyridoxal 5 phosphate, 1 mM pyruvate, and 0.5 mM ascorbic acid with or without 3 mM KYN (as substrate) and 2 mM aminooxyacetic acid (AOAA) (as non-specific aminotransferase inhibitor). The reaction was stopped by adding 30 μl 50% TCA and incubating on ice for 5 min. The reaction mixture was then centrifuged at 15 000*g* for 10 min, and the supernatant was collected for kynurenic acid (KYNA) analysis by HPLC. Blanks without placental homogenates were also analyzed to determine the non-enzymatic production of KYNA [41]. KAT activity was calculated as the difference between the amounts of KYNA produced in tubes with and without KYN after subtracting the corresponding blank values and is expressed as nanomoles of KYNA per milligram of protein per hour.

### Western blot analysis

Aliquots of placental homogenate (35 μg total protein) were mixed with loading buffer under reducing conditions [42], heated at 96°C for 5 min, and separated by SDS-PAGE on 10% (MAO) or 15% (IDO, KMO, KAT1, and TPH) polyacrylamide gels. Electrophoresis was performed at 120 V, and the separated proteins were transferred to a PVDF membrane (BioRad, Hercules, CA, USA). The membranes were blocked with a mixture containing 20 mM Tris-HCl pH 7.6, 150 mM NaCl, 0.1% Tween 20 (TBS-T), and 5% bovine serum albumin (BSA) for 1 h at room temperature and washed with TBS-T buffer. Incubation with primary antibodies was performed overnight at 4°C against MAO-A (Abcam, ab126751, dilution 1:1000), IDO (ThermoFisher Scientific, PA5-79437, dilution 1:1000), KMO (Proteintech, 10698-1-AP, dilution 1:1000), KAT1 (Proteintech, 12156-1-AP, dilution 1:500), and TPH (Invitrogen, PAI-777, dilution 1:100). After washing with TBS-T buffer, the membranes were incubated with the specific secondary antibody, i.e., anti-rabbit horseradish peroxidase-linked antibody (Dako, P0217, dilution 1:20 000), for 1 h at room temperature. Membranes were developed using the Chemiluminescence HRP Substrate Kit (ECL Prime Western Blotting System). Band intensity was visualized and quantified by densitometric analysis using the ChemiDoc MP, Imaging system (BioRad, Hercules, CA, USA). To ensure equal loading of proteins, membranes were probed for β-actin (Abcam, ab 8226, dilution 1:10 000) with anti-mouse HRP (Dako, P0260, dilution 1:20 000) as the specific secondary antibody.

### Analysis of TRP, 5-OH-TRP, 5-HT, 5-HIAA, KYN, KYNA, and QUIN

Concentrations of TRP, 5-OH-TRP, 5-HT, 5-HIAA, KYN and KYNA in cell-free supernatants were determined using a Shimadzu LC20 Performance HPLC chromatograph (Shimadzu, JP), as described previously [14]. A Kinetex EVO C18 100 A 150 × 3 mm, particle size 5 μm column (Phenomenex, USA) with a guard column was used at temperature of 20°C and a flow of 0.5 ml/min. 5-OH-TRP, 5-HT and TRP were eluted with a mobile phase consisting of 3:97 (v/v) methanol:acetic acid (0.1 M, pH 4.5, adjusted with NaOH). The excitation and emission wavelengths of the fluorescence detector were set at 276/333 nm. A mobile phase of 7:93 (v/v) methanol:acetic acid (0.2 M) with the same fluorescence detection wavelengths was used for the analysis of HIAA. Kynurenine was determined using a mobile phase consisting of 2:98 (v/v) methanol:acetic acid (0.1 M, pH 6.8, adjusted with NaOH) with UV detection at 289 nm. A mobile phase of 97:3 (v/v) zinc acetate (0.05 M with 0.025% acetic acid):acetonitrile was used for KYNA with excitation and emission wavelengths of 330/385 nm. Levels of QUIN were determined by a GC-MS method using deuterated quinolinic acid [43].

### Statistical analysis

Experimental outcomes were assessed using the non-parametric Mann–Whitney (when comparing two groups) or Kruskal–Wallis tests, followed by Dunn’s multiple comparisons test (when comparing more than two groups). The Kruskal–Wallis test was employed for statistical analysis of viability, cytokine levels, and qPCR data, while the Mann–Whitney test was employed for the assessment of key TRP metabolism enzymes by ddPCR, western blot, functional analysis, and metabolite release data. All analyses were implemented in GraphPad Prism 8.3.1 software (GraphPad Software, Inc., San Diego, USA). Asterisks in figures indicate significance levels: ^*^(*p* ≤ 0.05), ^*^^*^(*p* ≤ 0.01), ^*^^*^^*^(*p* ≤ 0.001), and ^*^^*^^*^^*^(*p* ≤ 0.0001).

## Results

### LPS and poly I:C induce gene expression and protein release of pro-inflammatory cytokine by placenta explants

**Figure 2:**
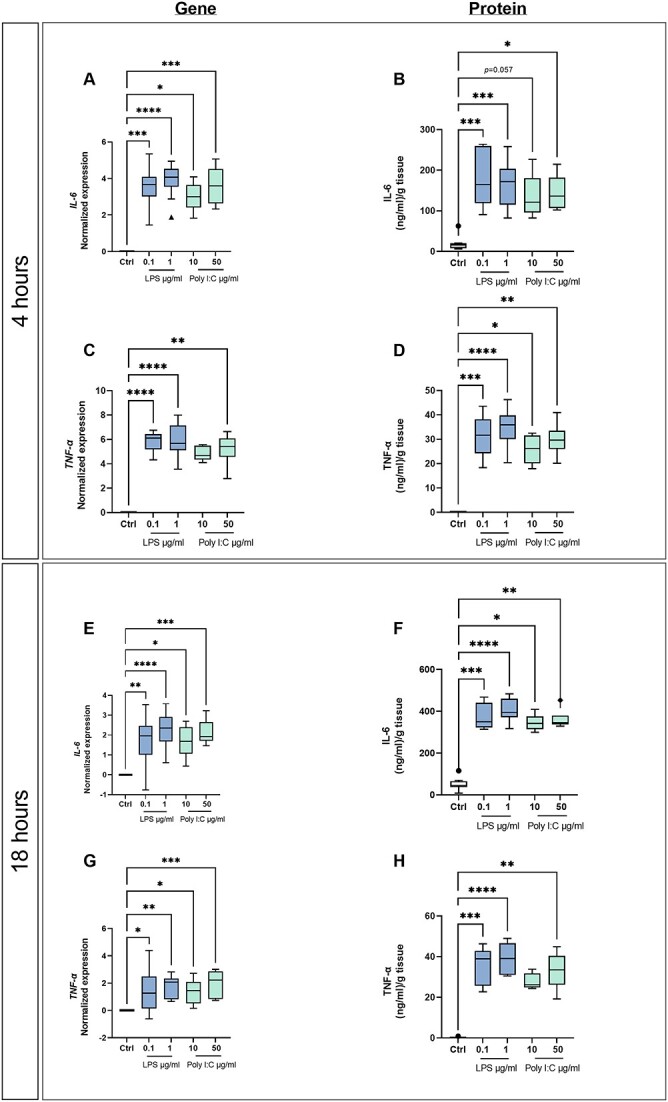
Gene expression and protein levels of pro-inflammatory cytokines in human term placenta explants exposed to LPS or poly I:C. Gene expression of *IL-6* (A, E) and *TNF-a* (C, G) from explants cultured for 4 h (A, C) or 18 h (E, G) with or without LPS or poly I:C was analyzed by qPCR. The release of IL-6 (B, F) and TNF-a (D, H) into the culture media by villous explants cultured for 4 h (B, D) or 18 h (F, H) with or without LPS or poly I:C was determined by ELISA. Cytokine concentrations in the conditioned media were corrected for the wet weight of the explant tissue. Data are presented as Tukey boxplots (1.5-times IQR); *n* ≥ 8. Statistical significance was evaluated using the non-parametric Kruskal–Wallis test, followed by Dunn’s multiple comparisons test; ^*^(*p* ≤ 0.05), ^*^^*^(*p* ≤ 0.01), ^*^^*^^*^(*p* ≤ 0.001), and ^*^^*^^*^^*^(*p* ≤ 0.0001).

Exposure of term placenta explants to LPS (0.1 and 1 μg/ml) or poly I:C (10 and 50 μg/ml) significantly increased gene expression of the pro-inflammatory cytokines IL-6 and TNF-α after 4 and 18 h incubations, as shown in Figure 2A, C, E, and G. Furthermore, the release of IL-6 and TNF-α cytokines into the culture media was significantly increased in explants exposed to LPS or poly I:C. Specifically, exposure to 0.1 μg/ml LPS for 4 and 18 h increased IL-6 release roughly 10-fold and 11-fold, respectively, when compared to controls. The corresponding fold increases when using an LPS concentration of 1 μg/ml were 11 and 13, respectively. Similarly, the IL-6 release following treatment with poly I:C increased roughly 4.5-fold after exposure for 4 h and 9.5-fold after 18 h, independently of the poly I:C concentration (10 or 50 μg/ml) (Figure 2B and F). The release of TNF-α also increased 410-fold and 500-fold after exposure to 0.1 μg/ml LPS for 4 and 18 h, respectively. The corresponding increases when using 1 μg/ml LPS were 480-fold and 580-fold, respectively. Finally, exposing explants to 10 or 50 μg/ml poly I:C for 4 h increased the release of TNF-α 350-fold and 400-fold, while 18 h of exposure increased TNF-α release 450-fold and 500-fold, respectively (Figure 2D and H).

### Placental explants secrete varying levels of TRP metabolites in response to LPS or poly I:C

**Figure 3:**
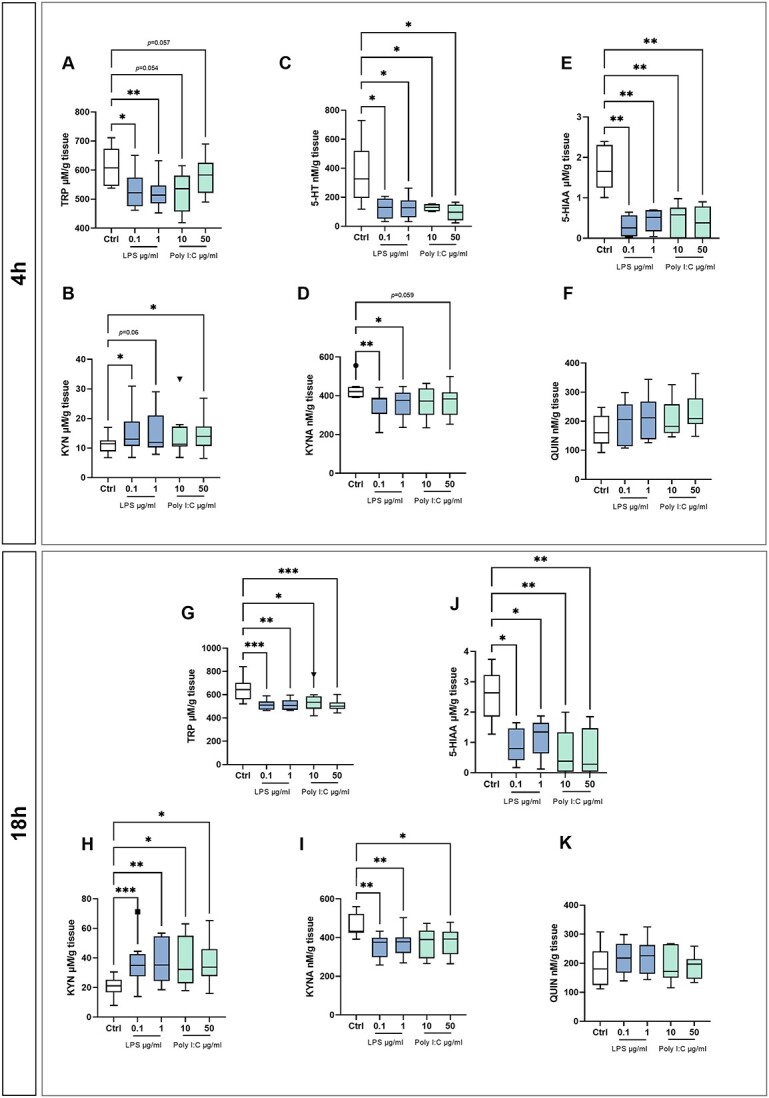
TRP metabolites in culture media from placenta explants exposed to LPS or poly I:C. Concentrations of TRP (A, G) and its main metabolites, serotonin (5-HT) (C), 5-hydroxyindoleacetic acid (5-HIAA) (E, J), KYN (B, H), KYNA (D, I), and QUIN (F, K) were evaluated in culture media from placental explants exposed to LPS (0.1 and 1 μg/ml) or poly I:C (10 and 50 μg/ml) for 4 (A–F) or 18 h (G–K). The results are reported using Tukey boxplots (1.5-times IQR) of metabolite concentrations normalized against the wet weight of the explant tissue *n* ≥ 8. Statistical significance was evaluated using the non-parametric Mann–Whitney test; ^*^(*p* ≤ 0.05), ^*^^*^(*p* ≤ 0.01), and ^*^^*^^*^(*p* ≤ 0.001).

We evaluated the impact of bacterial endotoxin (LPS) or viral (poly I:C) infection on the synthesis and secretion of TRP metabolites, including KYN, KYNA, QUIN, 5-HT, and 5-HIAA, by placental explants. The TRP concentration decreased significantly after treatment with LPS or poly I:C independently of the concentration used at every time point studied (Figure 3A and G), suggesting that placental explants consumed TRP that was present in the culture media and produced significant quantities of metabolites as shown in Figure 3. The KYN concentration in the culture medium was significantly higher for explants treated with LPS and poly I:C than for controls (Figure 3B and H), whereas the KYNA concentration declined significantly after treatment (Figure 3D and I). Treatment with LPS or poly I:C also caused the QUIN concentration to increase non-significantly compared to controls (Figure 3F and K). Furthermore, the KYN/TRP ratio in culture media from treated explants was significantly higher than that for controls (Table 1), indicating that TRP metabolism by the KYN pathway increased after exposing explants to LPS or poly I:C. However, the KYNA/KYN ratio decreased after treatment (Table 1), suggesting a decrease in activity along the aminotransferase (KAT) metabolic branch leading to production of the neuroprotective metabolite KYNA. Moreover, the QUIN/KYNA ratio increased significantly after 18 h treatments, suggesting a shift toward QUIN production instead of KYNA (Table 1).

**Table 1:** Ratios of tryptophan metabolites in culture media from human placental explants exposed to bacterial (LPS) and viral (poly I:C) infection. Results are expressed as medians (IQR); *n* = 8

Ratio	Time (h)	Control	LPS μg/ml		Poly I:C μg/ml	
			0.1	1	10	50
KYN/TRP ×10^3^	4	17.14 (13.64–21.06)	27.73 (23.01–34.85)^*^^*^^*^^*^	27.99 (19.81–42.18)^*^^*^	20.27 (18.32–34.84)	22.41 (20.04–30.69)^*^
	18	31.28 (24.54–44.43)	66.97 (59.21–82.75)^*^^*^^*^	74.84 (49.12–95.29)^*^^*^^*^	59.43 (42.53–105.0)^*^^*^	64.27 (53.07–93.12)^*^^*^^*^
KYNA/KYN ×10^3^	4	36.34 (32.13–46.55)	23.52 (19.59–31.78)^*^^*^	24.46 (20.69–31.36)^*^^*^	24.69 (21.11–40.28)	27.72 (18.47–34.31)^*^
	18	22.47 (18.61–34.48)	10.91 (9.3–16.34)^*^^*^^*^	9.79 (7.67–14.89)^*^^*^	13.13 (6.857–20.76)^*^	10.26 (7.90–16.39)^*^^*^
QUIN/KYN ×10^3^	4	67.01 (43.86–89.84)	71.34 (58.73–102.7)	66.44 (50.76–103.7)	61.93 (35.58–106.3)	93.89 (47.49–165.7)
	18	35.00 (24.82–57.43)	32.00 (16.23–54.44)	30.02 (19.20–60.31)	26.44 (19.62–36.66)	21.83 (16.92–40.47)
QUIN/KYNA ×10^3^	4	440.3 (347.8–553.9)	570.0 (528.5–786.0)	496.2(418.3–641.2)	491.7 (392.5–545.3)	540.3 (409.2–910.4)
	18	393.4 (233.3–463.8)	565.8 (509.0–595.5)^*^^*^	615.2 (445.7–801.5)^*^^*^	499.3 (410.5–610.7)	510.3 (430.9–569.8)^*^
5-HT/TRP ×10^3^	4	0.66 (0.39–0.90)	0.26 (0.10–0.30)^*^^*^	0.23 (0.12–0.34)^*^	0.22 (0.18–0.33)^*^	0.17 (0.07–0.24)^*^
HIAA /5-HT	4	4.58 (2.65–7.15)	4.78 (3.91–8.58)	4.94 (3.82–10.06)	4.92 (3.86–8.53)	6.96 (2.9–19.32)

Statistical significance was evaluated using the nonparametric Mann–Whitney test; ^*^(*p* ≤ 0.05), ^*^^*^(*p* ≤ 0.01), ^*^^*^^*^(*p* ≤ 0.001), and ^*^^*^^*^^*^(*p* ≤ 0.0001).

Production of 5-HT and 5-HIAA was significantly lower in placental explants stimulated with LPS or poly I:C than in untreated control cultures (Figure 3C, E, and J), indicating that exposure to LPS or poly I:C reduces the conversion of TRP via the 5-HT pathway. It should be noted that 5-HT was not detected in the culture media after 18 h of culture and was thus presumably metabolized by MAO during the longer incubations. Finally, the 5-HT/TRP ratio in media from explants treated for 4 h with LPS or poly I:C was significantly lower than in controls but the HIAA/5-HT ratio was unaffected (Table 1).

### Exposure to LPS or poly I:C regulates the relative gene expression of enzymes and transporters involved in TRP metabolic pathways in the human placenta

**Figure 4:**
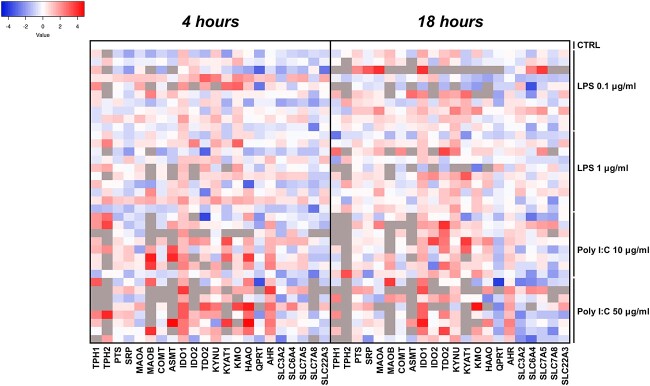
Gene expression of key enzymes and transporters involved in TRP metabolism in human placenta explants exposed to LPS or poly I:C. The heatmap shows fold changes in gene expression in placental explants incubated for 4 or 18 h in the presence and absence of LPS or poly I:C.

The effects of exposure to LPS (0.1 and 1 μg/ml) or poly I:C (10 and 50 μg/ml) for 4 or 18 h on the expression of 24 genes associated with TRP metabolism and transport in human placenta explants were evaluated using qPCR. Gene (mRNA) expression patterns were visualized using a heatmap as shown in Figure 4. The Kruskal–Wallis test revealed 11 genes whose expression differed significantly between control explants and those exposed to LPS or poly I:C. The KYN pathway was more strongly affected: treatment with LPS and/or poly I:C significantly increased the relative expression of *IDO1, TDO2, KYNU*, and *KMO* while reducing that of *QPRT*. The relative expression of four 5-HT pathway genes (*MAOA, MAOB, THP2*, and *PTS*) was also significantly upregulated following treatment with LPS or poly I:C, while the transport protein genes *SLC6A4* and *SLC7A8* were downregulated in explants treated with LPS and poly I:C or LPS alone, respectively. Interestingly, the aryl hydrocarbon receptor (AhR) gene was upregulated in explants exposed to poly I:C.

### Exposure to LPS or poly I:C shifts tryptophan metabolism toward the kynurenine pathway in the human term placenta

The levels of metabolite secretion and the relative gene expression of enzymes involved in TRP metabolism both differed significantly between control explants and those treated with LPS or poly I:C. For further investigation, ddPCR, western blotting, and enzymatic activity assays were used to evaluate the effect of LPS and poly I:C treatment on the gene and protein expression and functional activity of the main enzymes in the KYN pathway (IDO, KAT, and KMO) and the 5-HT pathway (TPH and MAO).

**Figure 5:**
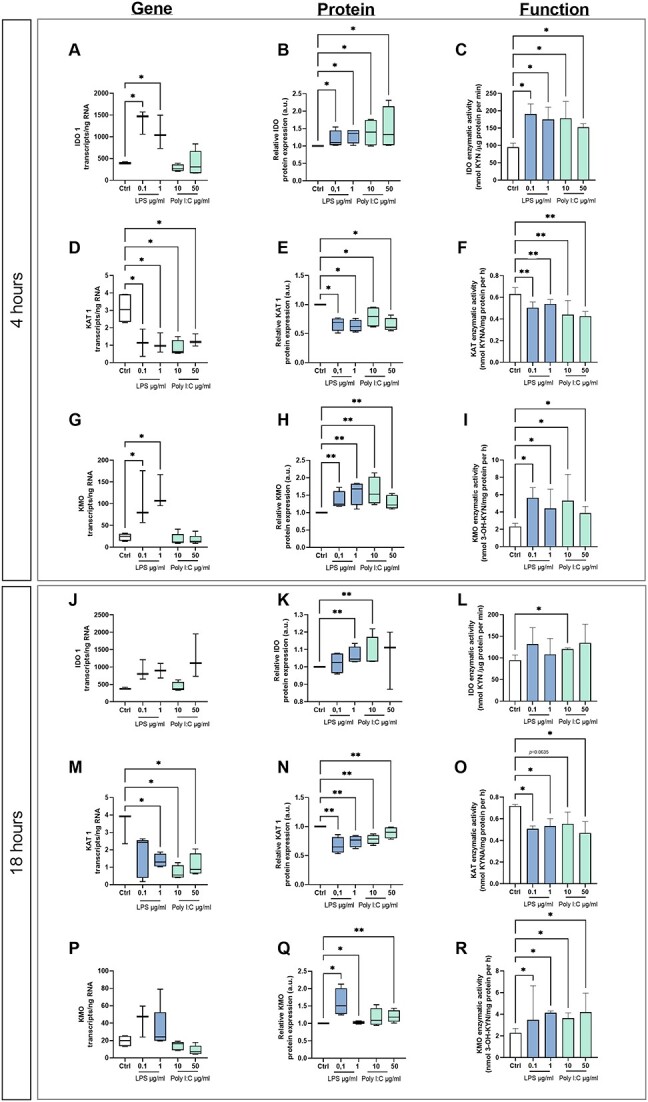
Gene/protein expression and functional analyses of the main enzymes of TRP metabolism via the KYN pathway in human placenta explants exposed to LPS or poly I:C. Villous explants were cultured for 4 h (A, D, G, B, E, H, C, F, I) or 18 h (J, M, P, K, N, Q, L, O, R) with or without LPS or poly I:C. Absolute quantitation of transcript numbers was achieved by ddPCR (A, D, G, J, M, P), and protein expression was evaluated by western blot analysis (B, E, H, K, N, Q). Protein expression was normalized against β-actin as a loading control. The enzymatic activity of IDO (C, L), KAT (F, O), and KMO (I, R) was evaluated as described in the Methods section. Data are presented as Tukey boxplots (1.5-times IQR) or medians with IQRs; *n* = 5. Statistical significance was evaluated using the non-parametric Mann–Whitney test; ^*^(*p* ≤ 0.05) and ^*^^*^(*p* ≤ 0.01).

**Figure 6:**
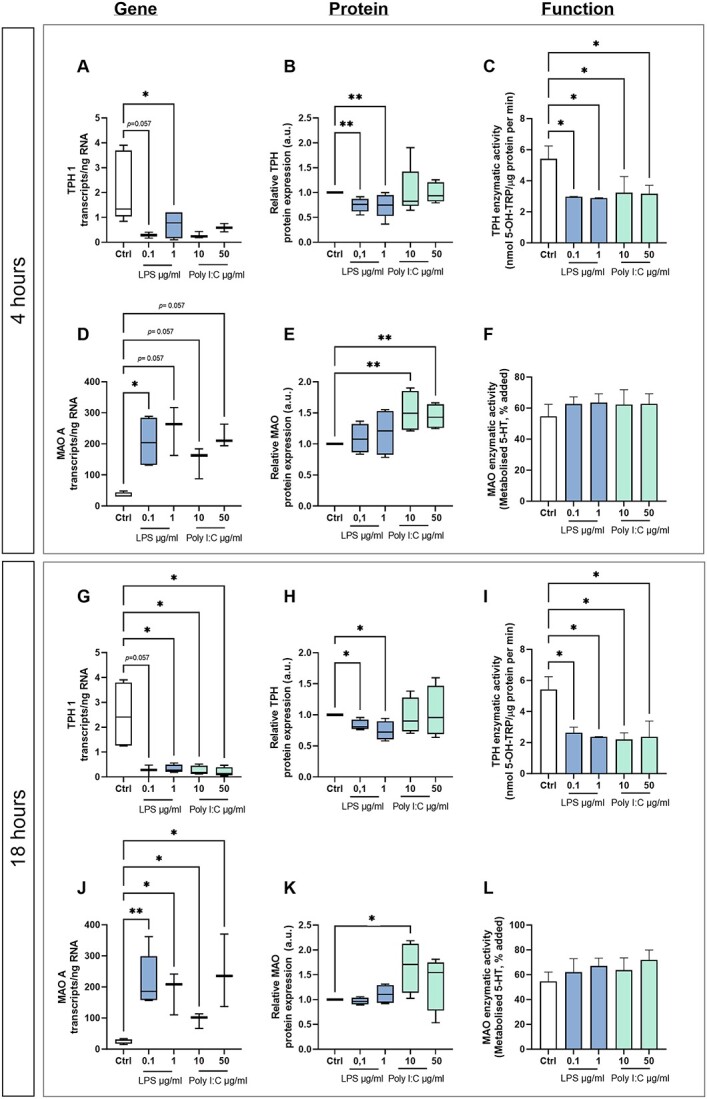
Gene/protein expression and functional analyses of the main enzymes of TRP metabolism via the 5-HT pathway in human placenta explants exposed to LPS or poly I:C. Villous explants were cultured for 4 h (A, D, B, E, C, F) or 18 h (G, J, H, K, I, L) with or without LPS or poly I:C. Absolute quantification of the number of transcripts was evaluated by ddPCR (A, D, G, J), and protein expression was evaluated by western blot analysis (B, E, H, K). Protein expression was normalized to β-actin as a loading control. The enzymatic activity of TPH (C, I) and MAO (F, L) was evaluated as described in the Methods section. Data are presented as Tukey boxplots (1.5-times IQR) or medians with IQRs; *n* = 5. Statistical significance was evaluated using the non-parametric Mann–Whitney test; ^*^(*p* ≤ 0.05) and ^*^^*^(*p* ≤ 0.01).

Absolute quantification of *IDO1*, *KAT1*, *KMO*, *TPH*, and *MAOA* transcripts in placenta explants cultured with and without LPS (0.1 and 1 μg/ml) or poly I:C (10 and 50 μg/ml) for 4 or 18 h was performed using ddPCR. The first and rate-limiting enzyme of the KYN pathway, *IDO1*, was upregulated after 4 h treatment with 0.1 or 1 μg/ml LPS (Figure 5A). However, exposure to LPS for 18 h (Figure 5J) or treatment with poly I:C (Figure 5A and J) had no significant effect on the number of *IDO1* transcripts. Similar results were observed for *KMO* (Figure 5G and P), whereas *KAT1* gene expression was downregulated after treatment with LPS or poly I:C after both 4 and 18 h of exposure (Figure 5D and M). Additionally, we observed statistically significant downregulation of *THP1*, the first and rate-limiting enzyme of the 5-HT pathway, in placental explants incubated with 0.1 and 1 μg/ml LPS for 4 h or with LPS or poly I:C for 18 h (Figure 6A and G). In contrast, *MAO-A* expression was upregulated after treatment with LPS or poly I:C for 4 or 18 h (Figure 6D and J).

To investigate changes in protein expression, quantitative western blot analyses using specific antibodies for IDO1, KAT1, KMO, TPH, and MAOA were performed using homogenates from placenta explants cultured with and without LPS (0.1 and 1 μg/ml) or poly I:C (10 and 50 μg/ml) for 4 or 18 h. Indoleamine 2,3-dioxygenase and KMO protein expression in explants exposed to LPS or poly I:C for 4 or 18 h was significantly higher than in controls (Figure 5B, H, K, and Q). Conversely, KAT1 protein expression fell after treatment with LPS or poly I:C (Figure 5E and N). In accordance with the gene expression data, TPH protein expression was reduced in placenta explants incubated with 0.1 and 1 μg/ml LPS for 4 and 18 h (Figure 6B and H), while MAO protein expression increased after poly I:C treatment for 4 or 18 h (Figure 6E and K).

Finally, we assessed the enzymatic activity of the selected proteins. This revealed that IDO activity increased significantly relative to controls following treatment with LPS and poly I:C for 4 h, independently of their concentration. However, longer exposure (18 h) to LPS or 50 μg/ml poly I:C had no significant effect on IDO activity (Figure 5C and L). Kynurenine monooxygenase activity increased in explants exposed to LPS or poly I:C, independently of the concentration and treatment duration (Figure 5I and R). Additionally, treatment with LPS or poly I:C significantly reduced the activity of both KAT (Figure 5F and O) and TPH (Figure 6C and I) relative to controls, independently of the treatment duration or applied concentration. However, MAO activity was unaffected by treatment with LPS or poly I:C (Figure 6F and L).

The gene expression, protein expression, and functional analysis results collectively indicate that exposition to LPS or poly I:C causes a shift in placental TRP metabolism from the 5-HT to the KYN pathway. Furthermore, within the KYN pathway, there is a preference for the KAT branch.

## Discussion

Despite strong epidemiological and experimental evidence of an association between prenatal inflammation and adverse neurobehavioral outcomes in exposed offspring [44], the mechanistic links between these processes are poorly understood. In this study, we used an ex vivo model of human placenta explants exposure to LPS and poly I:C to evaluate dysregulation of TRP metabolism and obtain additional insights into the mechanisms by which intrauterine infection affects placenta endocrine functions.

Inflammation is the natural response of the body to harmful stimuli, like pathogens, injured or deceased cells, toxins, or radiation [45]. In our study, we restrict the inflammation process to a complex cascade of immune responses to LPS or poly I:C treatment, encompassing various molecular and cellular processes [46]. The mammalian immune system recognizes LPS and poly I:C via Toll-like receptors 4 and 3, respectively. This recognition triggers the up-regulation, production, and release of many pro-inflammatory cytokines, including IL-6 and TNF-α [47]. In our model, exposing human placenta explants to LPS or poly I:C significantly increased the expression and secretion of the pro-inflammatory cytokines IL-6 and TNF-α, which is consistent with previous reports describing cytokine production in the human placenta [48]. Nevertheless, human placental explants consist of a heterogeneous cell population, including various cell types, such as syncytiotrophoblasts [49], cytotrophoblasts [50], Hoffbauer cells [51], and endothelial cells [52]; contribution of these cell types to the release of cytokines needs to be elucidated in further studies. It’s worth noting that while we observed the release of the specific pro-inflammatory cytokines, IL-6 and TNF-α, we cannot rule out the possibility that other cytokines are released in response to TLR4 and TLR3 activation. Importantly, the viability parameters of the treated explants, including LDH activity in the culture media and mitochondrial activity (evaluated using the MTT assay), did not differ from those of controls (Supplementary figure S1). This means that the applied concentrations of LPS or poly I:C did not compromise the integrity and viability of the explants, so the release of cytokines in our model was due to the LPS or poly I:C treatment rather than tissue damage.

The metabolism of the essential amino acid TRP is important in the regulation of immune responses, generation of oxidative radicals, and production of neuroregulatory substances [28, 53]. It is also highly sensitive to pro-inflammatory cytokines [54]. Accordingly, the concentration of TRP in culture media containing explants treated with LPS or poly I:C was lower than in controls, suggesting that TRP consumption increased following the activation of TLR4 and TLR3 receptors. Increased TRP metabolism under systemic inflammation has previously been reported in patients with neurovascular diseases [55], obesity [56], several types of cancer [57], traumatic brain injury [58], and pulmonary arterial hypertension [59], as well as in animal models of maternal immune activation [30, 60].

During an inflammatory response, the degradation of TRP via KYN pathways is increased by the release of pro-inflammatory cytokines, in particular IFN-γ, IL-6, and TNF-α [61]. These cytokines induce transcription of IDO, the rate-limiting enzyme in the catabolic transformation of TRP into KYN, in a variety of cells including placental cells [62]. Therefore, an increased IDO level could shift the balance of TRP metabolism toward KYN formation. In our study, human placenta explants stimulated with LPS or poly I:C exhibited increased gene and protein expression of IDO as well as increased IDO enzyme activity. Consequently, the concentration of KYN and the KYN/TRP ratio in the culture media both increased. Treatment with LPS or poly I:C also reduced the concentration of KYNA and the KYNA/KYN ratio in the explant culture medium, which can be explained by the reduced expression and functional activity of KAT in the explants exposed to LPS or poly I:C. Interestingly, KYNA was reported to have antioxidant properties and to mediate immunosuppressive effects under inflammatory conditions, reducing the expression and secretion of TNF-α and downregulating the IL23/IL17 axis [63]. Reductions in the concentration of KYNA may thus help maintain the pro-inflammatory state in the placenta [64].

Pro-inflammatory cytokines also activate KMO [65], which converts KYN into 3-OH-KYN and further shifts the KYN pathway toward the neurotoxic 3-OH-KYN/QUIN branch instead of the KYNA branch. Placental explants exposed to LPS or poly I:C exhibited increased gene and protein expression of KMO and elevated KMO enzymatic activity; this suggests that the enzyme in the human placenta is strongly affected by the release of pro-inflammatory cytokines upon the activation of TLR4 and TLR3 receptors, after the treatment with LPS and poly I:C. Despite this, the levels of 3-OH-KYN during our experiments were below the limit of detection; possibly, 3-OH-KYN is labile and rapidly converted into downstream metabolites. Accordingly, the KYNU gene was upregulated in explants exposed to LPS or poly I:C, suggesting an increase in the conversion of 3-OH-KYN into 3-hydroxyanthranilinic acid en route to the production of QUIN. Interestingly, the mRNA expression of QPRT, the enzyme responsible for QUIN clearance, reduced after 18 h treatment with LPS or poly I:C, indicating that the increased production of QUIN may be accompanied by a reduction in its rate of metabolic degradation. In keeping with this hypothesis, increased levels of QUIN were detected in placenta explants exposed to LPS for 48 h [66]. We also observed that the concentration of QUIN in the supernatant medium tended to increase in explants exposed to LPS or poly I:C.

Importantly, we found that the QUIN/KYNA ratio in explants exposed to LPS or 50 μg/ml poly I:C for 18 h was significantly higher than in controls, indicating a shift toward enhanced QUIN formation and reduced KYNA production. Kynurenic acid exerts a neuroprotective effect by inhibiting the α7 nicotinic acetylcholine, N-methyl-D-aspartate (NMDA), and kainite glutamate receptors [67]. Conversely, QUIN promotes lipid peroxidation and has neurotoxic activity due to activation of NMDA receptors. The pro-inflammatory cytokine IFN-γ increases QUIN levels in the human brain [68], and QUIN production has been linked to the pathogenesis of many neurological conditions including depression and schizophrenia [69]. Moreover, an imbalance between QUIN and KYNA was observed in human serum after systemic LPS injection [70]. Finally, increased QUIN concentrations in cerebrospinal fluid are strongly associated with mortality in children and adults after traumatic brain injury [71, 72] and the QUIN/KYNA ratio has been suggested as a biomarker for post-stroke cognitive decline [73]. The increased QUIN/KYNA ratio observed in our study may thus indicate the development of a neurotoxic environment during placental infections.

Placenta explants exposed to LPS or poly I:C exhibit clear dysregulation of the KYN pathway, which could potentially lead to the release of neuroactive metabolites into the fetal circulation [66]. While the maternal–fetal transport of KYN metabolites has been poorly studied, concentrations of TRP and KYN metabolites in cord blood are reportedly higher than in maternal blood, indicating active transport from a mother to her fetus [74]. Under physiological conditions, there is a significant transplacental transfer of KYN but that of KYNA is less pronounced [18]. Moreover, increased QUIN concentrations have been observed in umbilical cord venous blood during pregnancies with intrauterine infections [31]. Impairment of the placental KYN pathway due to elevated pro-inflammatory cytokines may thus expose the fetus to potentially neurotoxic substances such as QUIN. Since the placenta explant technique does not enable us to distinguish the release of metabolites into the maternal or fetal circulations, further studies on the transport of KYNA and QUIN from the placenta to the fetal circulation are needed to determine the overall importance of this process.

Our findings are consistent with previous reports demonstrating that infectious agents upregulate the expression of several enzymes of the KYN pathway, including IDO, TDO, KYNase, and 3HAO in placental tissues challenged with intrauterine infections in vivo or following exposure to the bacterial endotoxin LPS in vitro [31]. Additionally, our group recently identified impairments in KYN pathway gene expression in placentas from preterm birth pregnancies that were positively associated with maternal inflammatory markers [32]. However, changes in mRNA expression do not necessarily reflect alterations in enzyme activation and function [75, 76]. Importantly, our study offers a robust approach by comprehensively examining both gene and protein expressions alongside protein function to deepen our understanding of TRP metabolism following the treatment with LPS or poly I:C.

Experimental evidence suggests that gestational 5-HT dysregulation adversely affects fetal brain development and is associated with the pathogenesis of neurodevelopmental disorders [77, 78]. Explants exposed to LPS or poly I:C exhibited reduced 5-HT levels and 5-HT/TRP ratios, suggesting a decrease in TRP metabolism by the 5-HT pathway. This could be a consequence of the activation of placental IDO, which would reduce the availability of TRP to placental TPH. However, we observed downregulation of TPH expression and enzymatic activity in our model, indicating an effect of LPS and poly I:C treatment on TPH. Previous studies have found that intrauterine inflammation, triggered by intrauterine endotoxin administration, reduces 5-HT levels in the placenta and brains of newborn rabbits [30, 79]. On the other hand, maternal inflammation, initiated by the systemic administration of poly I:C, upregulates 5-HT synthesis in the murine placenta [29]. Furthermore, xanthurenic acid, a metabolite formed from 3-OH-KYN after activation of the KYN pathway, was shown to inhibit the final enzyme in de novo BH4 synthesis, sepiapterin reductase, and to thereby reduce the levels of BH4, which is required for the catalytic cycle of TPH [80]. Reduced levels of BH4 could therefore also reduce 5-HT production. Interestingly, the 5-HT-metabolizing enzyme MAO was upregulated in placenta explants exposed to LPS or poly I:C, suggesting that the rate of metabolic degradation of 5-HT increased while that of its synthesis was reduced.

We have previously shown that under physiological conditions, TRP metabolism in the placenta is a dynamic process that changes during gestation based on fetal requirements [14, 15]. Therefore, the effects of an inflammatory challenge on the fetus depend strongly on its timing. In the early stages of pregnancy, the fetus depends on placental 5-HT synthesis for the development of 5-HT-dependent organs, including the brain. A decrease in 5-HT production due to inflammatory insults during this stage could thus have deleterious effects on fetal brain development. Conversely, in the final stages of pregnancy, the fetus can synthesize its own 5-HT from maternal TRP. However, increased levels of pro-inflammatory cytokines reduce the availability of TRP and thus decrease its levels in the fetus, with consequences for 5-HT production in the fetal brain. Similarly, a decrease in 5-HT concentrations has been observed in the brains of neonatal rabbits after in utero exposure to *E. coli* endotoxin [79].

The decrease in 5-HT production in the placenta due to inflammatory stimuli may affect levels of melatonin, a downstream metabolite in the 5-HT pathway with antioxidant and anti-inflammatory properties. Melatonin has been reported to reduce the production of cytokines associated with inflammation [81], so any reduction in its concentration could help maintain a pro-inflammatory state in the placenta. Furthermore, melatonin was postulated to play a role in several neurological diseases with inflammatory components including dementia, Alzheimer’s disease, and Parkinson’s disease [82].

We observed some discrepancies between the LPS and poly I:C treatments even though they both increased the concentrations of pro-inflammatory cytokines (IL-6 and TNF-α). Remarkably, inflammation induces an increase in AhR expression, a transcriptional factor that controls local and systemic immune responses. AhR is activated by its ligand, KYN, and induces the secretion of anti-inflammatory cytokines such as IL-10. Interestingly, explants treated with poly I:C exhibited upregulated AhR gene expression, suggesting that poly I:C may activate AhR and trigger the production of anti-inflammatory cytokines. Further experiments are needed to investigate this hypothesis.

This study is limited to using term placentas, thus providing insights into TRP metabolism impairment only in the later stages of pregnancy. Admittedly, the immune system in pregnant women changes as gestation progresses [83], potentially leading to variations in cytokine responses. Additionally, placental TRP metabolism is a dynamic process that undergoes alterations throughout pregnancy [14, 15]. As a result, the response of placental TRP metabolism to inflammatory cytokines might differ during the earlier stages of pregnancy. This question remains to be investigated using appropriate experimental model(s).

## Conclusions

**Figure 7:**
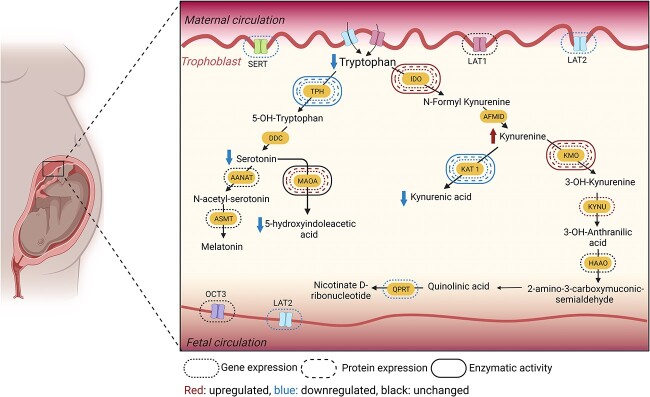
Schematic illustration of the modifications occurring in the placental TRP metabolic pathways after exposure to LPS or poly I:C. Created in BioRender.com.

Exposure of human placenta explants to LPS or poly I:C impairs TRP homeostasis in the human term placenta, which might eventually affect the programming of the fetus. This effect is mediated by decreased 5-HT production during intrauterine life, which may have a negative impact on fetal brain development. In addition, the imbalance in the QUIN/KYNA ratio, as a result of increased activity along the KMO branch of TRP metabolism, together with the release of KYN metabolites from the placenta, may expose the fetus to higher amounts of potentially neurotoxic molecules (Figure 7). However, it is crucial to underscore that the interplay between placental inflammation, characterized by an increase in pro-inflammatory cytokines, TRP metabolism, and fetal development, is intricate and multifaceted. Further studies are necessary to elucidate the precise mechanisms and underlying causative factors contributing to the observed changes.

## Supplementary Material

Supplementary_Figure_1_ioad181

Abad_et_al_2023_supplementary_ioad181

## Data Availability

All data generated or analyzed during this study are available from the corresponding author on reasonable request.
