# Supplementary figures and images for: Pathological shifts in tryptophan metabolism in human term placenta exposed to LPS or poly I:C
Source: Biol Reprod. 2023 Dec 25;110(4):722–38. doi: 10.1093/biolre/ioad181 (PMC11017130; doi:10.1093/biolre/ioad181)

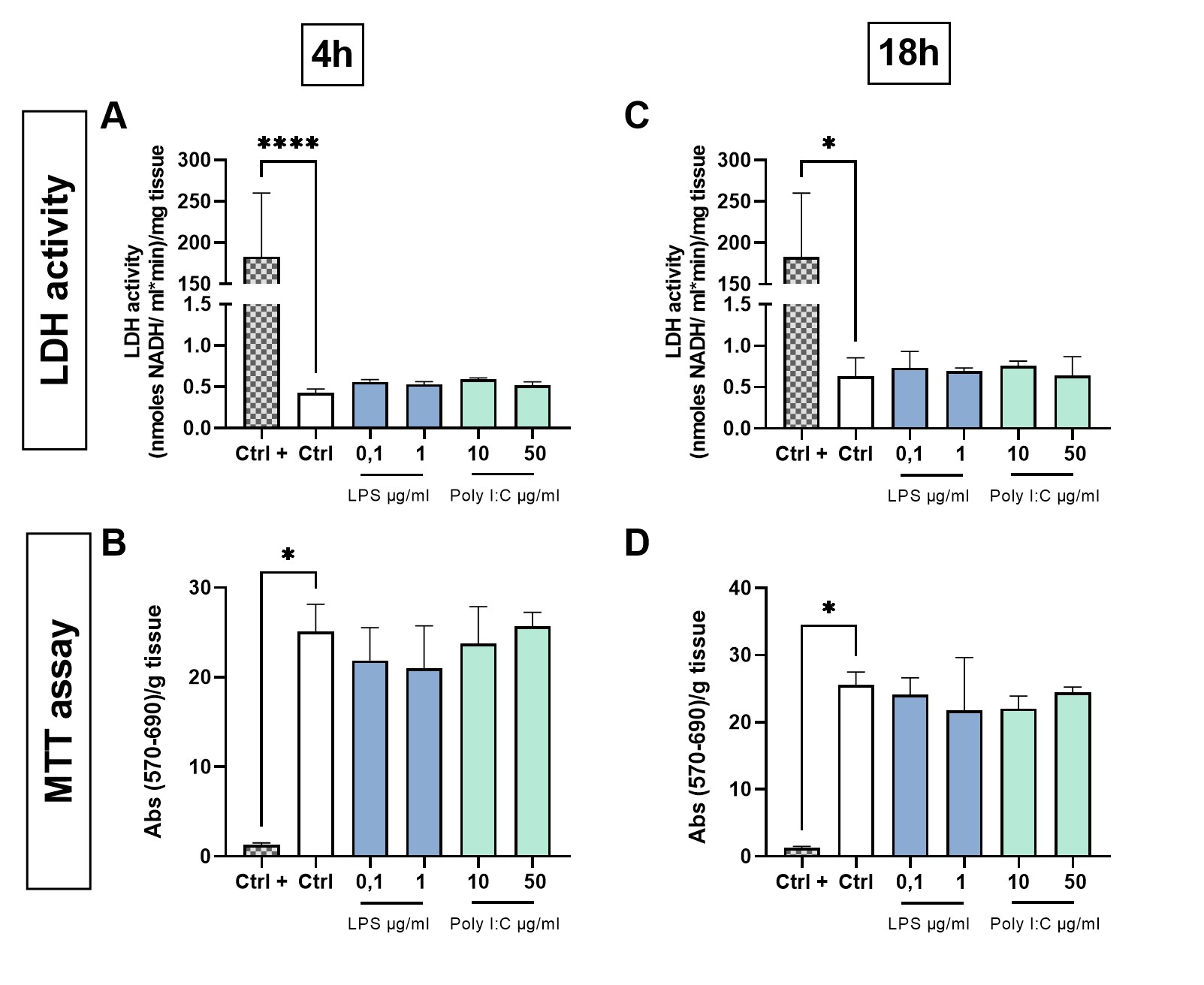

Supplement: Supplementary_Figure_1_ioad181 [file supplementary_figure_1_ioad181.jpeg]
