# Supplementary material for: Pathological shifts in tryptophan metabolism in human term placenta exposed to LPS or poly I:C
Source: Biol Reprod. 2023 Dec 25;110(4):722–38. doi: 10.1093/biolre/ioad181 (PMC11017130; doi:10.1093/biolre/ioad181)
Supplement: Abad_et_al_2023_supplementary_ioad181 [file abad_et_al_2023_supplementary_ioad181.docx]

Supplementary data:

**LPS and Poly I:C do not compromise the integrity and viability of the explants**

Cell membrane integrity was assayed by measuring the release of the intracellular enzyme LDH into the culture media after 4 h or 18 h of treatment with LPS or Poly I:C. There were no significant differences in LDH activity in culture supernatants from explants cultured with LPS (0.1 and 1 µg/ml) or Poly I:C (10 and 50 µg/ml) compared to controls (see Supplementary Figure 1A and 1C). To quantify the maximal activity of LDH in the culture media, explants were treated with lysis buffer for 15 min at 37 °C as positive controls. Similarly, the viability of placenta explants was evaluated during experiments by analyzing mitochondrial activity in the tissue using the MTT assay. No changes in mitochondrial activity relative to controls were observed in explants incubated for 4h (Supplementary Figure 1B) or 18 h (Supplementary Figure 1D) with LPS (0.1 and 1µg/ml) or Poly I:C (10 and 50 µg/ml). Explants cultured for 18 h in the presence of 40% DMSO were used as death controls (positive control).

**Supplementary Figure 1. Viability of human term placenta explants.** The integrity of the explants was assayed by measuring the activity of LDH in the culture media of explants cultured for 4 hours (A) or 18 hours (C) in the presence of LPS or Poly I:C. Explants treated with lysis buffer for 15 min at 37°C were used as positive controls (Ctrl+). The viability of the explants was evaluated using the colorimetric MTT assay, which measures mitochondrial activity. The incorporation of MTT was determined in explants cultured with and without LPS or Poly I:C for 4 hours (B) or 18 hours (D). Explants cultured with 40% DMSO for 18 hours were used as positive controls (Ctrl+). Data are presented as medians with IQRs; n = 5. Statistical significance was evaluated using the non-parametric Kruskal–Wallis test, followed by Dunn's multiple comparisons test; * (p ≤ 0.05) and **** (p ≤ 0.0001).
